# SARS‐CoV‐2 Omicron variants in Bangladesh: Pandemic to endemic

**DOI:** 10.1002/hsr2.1134

**Published:** 2023-03-02

**Authors:** Mohammad Jubair, Mst. Noorjahan Begum, Sezanur Rahman, Sourav Mohammad Arefeen Haider, Shovan B. Moon, Mohammad Enayet Hossain, Mohammed Ziaur Rahman, Manjur H. Khan, Ahmed N. Alam, Tahmina Shirin, Mokibul H. Afrad, Firdausi Qadri, Mustafizur Rahman

**Affiliations:** ^1^ Infectious Diseases Division International Centre for Diarrhoeal Disease Research (ICDDR) Mohakhali Dhaka Bangladesh; ^2^ Laboratory Sciences and Services Division International Centre for Diarrhoeal Disease Research (ICDDR) Dhaka Bangladesh; ^3^ Department of Virology, Institute of Epidemiology Disease Control and Research Dhaka Bangladesh

**Keywords:** COVID‐19, Omicron, protection, reinfection, SARS‐CoV‐2, XBB

## 1 INTRODUCTION

Several SARS‐CoV‐2 variants of concern (VOCs) have emerged and spread globally since 2021, including in Bangladesh.^1^ Omicron is the latest edition, first identified in South Africa in November 2021 and arrived in Bangladesh in December 2021. This variant frequently changed with different sublineages, especially BA.1, BA.2, BA.5, and XBB, replacing one by another over time.^2^, ^3^ The clinical consequence of these shifts has been less explored.^4^ As part of the countrywide COVID‐19 testing network, International Centre for Diarrheal Disease Research, Bangladesh (icddr,b) has been monitoring SARS‐CoV‐2 variants since January 2021.^5^ Here, we describe the emergence and comparative clinical manifestations in patients infected with different Omicron sublineages.

## 2 METHODS

Screening of SARS‐CoV‐2 variants was carried out with a subset of real‐time polymerase chain reaction (PCR) positive samples having cycle threshold values <30 by using complete genome sequencing through Illumina (MiSeq) platform.^1^, ^5^ Retrospective meta‐data on clinical manifestation were collected from individuals infected with different Omicron sublineages. Data were collected from 466 participants on a structured questionnaire through a phone call after getting verbal informed consent. The respondents were the patients themselves or relatives of the deceased patients who knew details about the patient's COVID‐19 illness‐related history. The moderate, severe, or asymptomatic diseases were determined according to the classification of World Health Organization and Bangladesh national guideline on COVID‐19 case management.^6^, ^7^ The individuals who had hypertension, diabetes mellitus, chronic cardiac disease, tuberculosis, chronic obstructive pulmonary disease, chronic pulmonary disease, ischemic heart disease (IHD), asthma, malignant disease, chronic kidney disease (CKD), immunodeficiency including HIV, chronic liver disease (CLD), chronic neurological disorder, and other comorbidity, if any, were considered as comorbid. Data on pre‐existing comorbidity were also collected and analyzed. However, we only selected hypertension and diabetes as significantly present in our comorbid individuals. Four different types of vaccines were used through Government network, for example, Pfizer‐BioNTech, Oxford‐AstraZeneca, Moderna, and Sinopharm BBIBP‐CorV. There was no vaccination record of three participants. The individuals, who were PCR positive for the second time, considered as reinfected. We collected new samples from the individual and ran new real‐time PCR to confirm reinfection. Statistical significance between the variables was tested by using *χ* ^2^ test.

## 3 RESULTS

**Figure 1:**
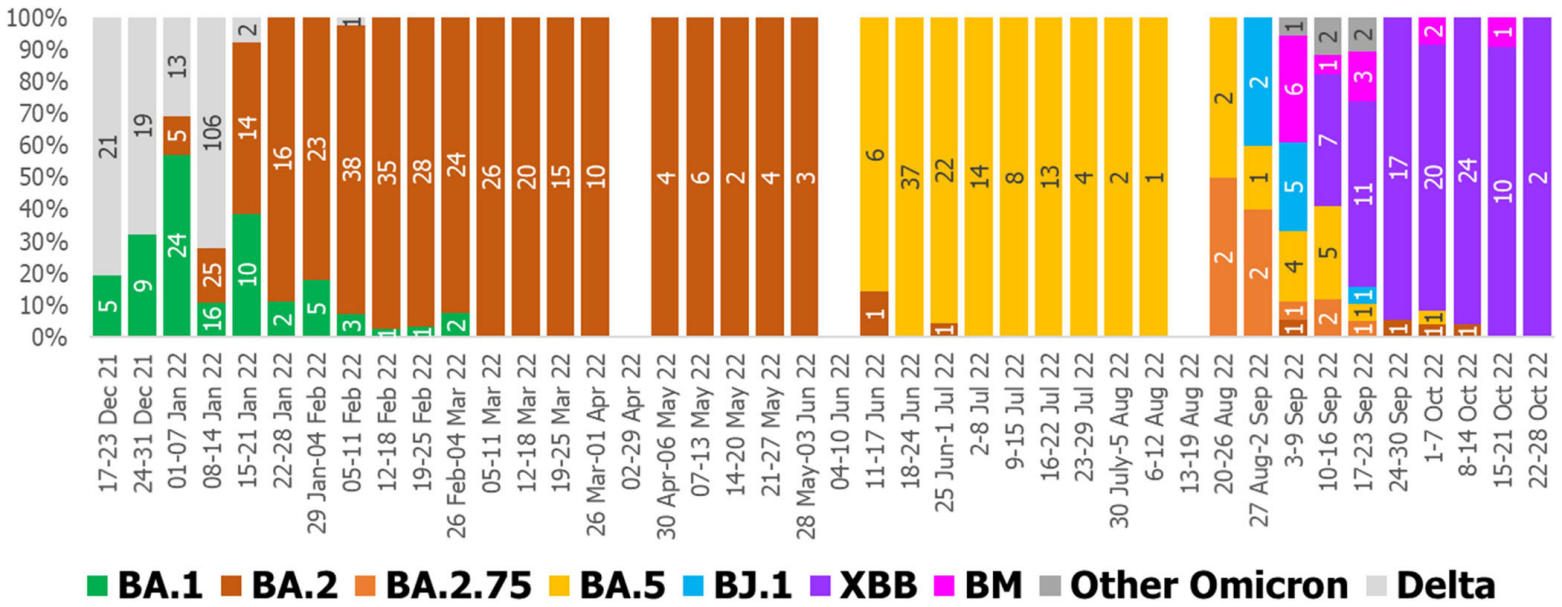
Weekly distribution of different Omicron sublineages in Bangladesh from December 2021 to October 2022.

From December 2021 to October 2022, we sequenced 575 Omicron variants belonging to at least seven sublineages. BA.1 arrived first and continued for a short period until February 2022 (Figure 1). In the meantime, BA.2 emerged in January 2022 and had been the most prevalent sublineage up to June 2022. BA.5 emerged in June 2022 and was established as the only circulating sublineage by replacing all the previous ones. Consequently, Omicron XBB appeared in September 2022 and became the most predominant sublineage in the country.

Among 466 individuals who provided the clinical data, 81 were infected with Omicron BA.1, 290 with BA.2, 63 with BA.5 and 32 with XBB. Difference between these sublineages for demographic variables, clinical manifestations (headache, body ache, weakness), disease severity (severe, mild to moderate), reinfection, and vaccination status (booster, fully vaccinated) of the patients was analyzed. The XBB infection was higher among 41–50 years (*p* = 0.043) and lower among 18–30 years (*p* < 0.001) as compared to other sublineages (Table 1). Overall severity of the Omicron sublineages was low; however, with limited number of patients, XBB showed more severe disease and caused more hospitalizations compared to others (*p* < 0.001). Headache (*p* = 0.01), body ache (*p* < 0.001), and weakness (*p* < 0.001) were more noticeable among the XBB‐infected individuals.

**Table 1:** Comparative analysis of available meta data for Omicron BA.1, BA.2, BA.5, and XBB sublianage.

Variables	BA.1 (*N* = 81), *n* (%)	BA.2 (*N* = 290), *n* (%)	BA.5 (*N* = 63), *n* (%)	XBB (*N* = 32), *n* (%)	*p* Value
Age groups (years)					
<5	0 (0%)	11 (4%)	0 (0%)	0 (0%)	‐
5–17	7 (9%)	16 (6%)	4 (6%)	1 (3%)	0.658
18–30	20 (25%)	67 (23%)	31 (49%)	3 (9%)	**<0.001**
31–40	17 (21%)	66 (23%)	16 (25%)	10 (31%)	0.666
41–50	11 (14%)	44 (15%)	6 (10%)	10 (31%)	**0.043**
51–60	16 (20%)	41 (14%)	3 (5%)	4 (13%)	0.077
>60	10 (12%)	45 (16%)	3 (5%)	4 (13%)	0.151
Sex					
Male	44 (54%)	162 (56%)	35 (56%)	18 (56%)	0.995
Female	37 (46%)	128 (44%)	28 (44%)	14 (44%)	0.995
Disease severity					
Severe	3 (4%)	11 (4%)	3 (5%)	6 (19%)	**<0.001**
Mild to moderate	78 (96%)	279 (96%)	52 (82%)	25 (78%)	**<0.001**
Asymptomatic	0 (0%)	0 (0%)	8 (13%)	1 (3%)	‐
Symptoms					
Fever	63 (78%)	189 (65%)	44 (70%)	21 (66%)	0.213
Cough	47 (58%)	155 (53%)	31 (49%)	16 (50%)	0.137
Headache	12 (15%)	39 (13%)	18 (29%)	14 (44%)	**0.007**
Body ache	6 (7%)	11 (4%)	15 (24%)	13 (41%)	**<0.001**
Runny nose	25 (31%)	92 (32%)	12 (19%)	12 (37%)	0.116
Weakness	17 (21%)	24 (8%)	17 (27%)	12 (37%)	**<0.001**
Comorbidity					
Comorbidity present	30 (37%)	84 (29%)	18 (29%)	17 (53%)	0.802
Hypertension	19 (63%)	40 (48%)	7 (39%)	7 (41%)	0.480
Diabetes	15 (50%)	35 (42%)	4 (22%)	1 (17%)	0.059
Hypertension and diabetes	9 (30%)	20 (24%)	2 (11%)	3 (14%)	0.497
Vaccination					
Fully vaccinated with booster	11 (14%)	37 (13%)	15 (24%)	21 (66%)	**<0.001**
Fully vaccinated	61 (75%)	204 (70%)	39 (62%)	8 (25%)	**<0.001**
No vaccine	9 (11%)	49 (17%)	6 (10%)	3 (9%)	0.237
Other meta‐data					
Hospitalized	3 (4%)	11 (4%)	3 (5%)	6 (19%)	**0.025**
Deaths	0 (0%)	8 (3%)	0 (0%)	3 (9%)	‐
Travel history	1 (1%)	12 (4%)	1 (2%)	4 (13%)	0.111
Reinfection	7 (9%)	23 (8%)	22 (35%)	15 (47%)	**<0.001**

*Note*: Bold values indicate statistical significant values between the variables.

## 4 DISCUSSION

This study depicts the emergence and evolution of the Omicron variants, which were circulating in Bangladesh when the majority of the population was vaccinated. Several sublineages of the variant appeared, disappeared and were replaced one by another over time. The overall disease severity of Omicron was minimum (5%) may be due to high vaccination coverage (86%) in this population (Table 1).

Most of the Omicron‐infected patients in our study had a history of natural infection or vaccination and had mild to moderate symptoms (95%). This indicates that pre‐existing immunity might not provide full protection against Omicron variant but minimize the severity. Molecular evidence of reinfection from previous studies indicated that genetically distinct strains or new variants could escape immunity.^8^, ^9^, ^10^ To combat the pandemic, the Bangladesh government started mass COVID‐19 vaccination in February 2021, and till now, 75% population completed the full dose and 86% of at least one dose.^11^ This reflects the countrywide COVID hospitalization scenario where most of the beds were vacant during the Omicron wave.^12^, ^13^ Although the overall severity of the Omicron variant was low, when compared among the Omicron sublineages, hospitalizations and deaths were higher for XBB infections, while the number of positive cases was lower compared to other Omicron sublineages. This descending trend was also observed during October to November 2022 when the countrywide COVID positivity rate was less than 1% which might be an indication entering of a pandemic virus into an endemic era.

The study has several limitations. First, meta‐data were collected retrospectively over a phone call, imposing a recall bias, especially for children and deceased cases where respondents were guardians or relatives. Although there was no refusal, some patients could not be reached over the phone, and their clinical features were unavailable. The number of sequence data presented here is very low compared to the total number of cases, which might fail to illustrate the true scenario of the Omicron wave.

In conclusion, our study illustrated how new variants outcompeted the previous ones and impacted by vaccination or natural infection. During last 1 year, there were over 500 sublineages of this variant circulating, but not one had been designated as a new VOC. The question is whether the Omicron is the latest variant or becomes more humanized to circulate as endemic. To answer this question, continuation of genomic surveillance for a regular update on the emergence of new variants is warranted.

## AUTHOR CONTRIBUTIONS

**Mohammad Jubair**: Conceptualization; data curation; formal analysis; supervision; validation; writing—original draft; writing—review and editing. **Mst. Noorjahan Begum**: Data curation; formal analysis; supervision; writing—original draft; writing—review and editing. **Sezanur Rahman**: Data curation; formal analysis; validation; visualization; writing—original draft; writing—review and editing. **Sourav Mohammad Arefeen Haider**: Formal analysis; methodology; writing—review and editing. **Shovan B. Moon**: Formal analysis; validation; writing—review—review and editing. **Mohammed Ziaur Rahman**: Investigation; supervision; valid—review and editing. **Ahmed N. Alam**: Investigation; supervision; writing—review and editing. **Tahmina Shirin**: Funding acquisition; investigation; supervision; writing—review and editing. **Mokibul H. Afrad**: Formal analysis; validation; writing—review and editing. **Firdausi Qadri**: Conceptualization; funding acquisition; investigation; methodology; supervision; validation; writing—review and editing. **Mustafizur Rahman**: Conceptualization; data curation; formal analysis; funding acquisition; investigation; methodology; project administration; supervision; validation; visualization; writing—original draft; writing—review and editing.

## CONFLICT OF INTEREST STATEMENT

The authors declare no conflict of interest.

## ETHICS STATEMENT

This study was approved by the icddr,b institutional ethical review committee. All meta‐data were collected after getting informed consent from the participants. The privacy and confidentially of respondents were strictly maintained at all steps of data collection, data management, and analysis. All personal identifiers (i.e., names, addresses and phone number) were removed before analysis.

## TRANSPARENCY STATEMENT

The lead author Mustafizur Rahman affirms that this manuscript is an honest, accurate, and transparent account of the study being reported; that no important aspects of the study have been omitted; and that any discrepancies from the study as planned (and, if relevant, registered) have been explained.

## Data Availability

All data and materials used in this work were publicly available. Sequences were published in GISAID (www.gisaid.org) database. All authors had full access to all the data in the study and accepted the responsibility to submit for publication
